# Supplementary material for: Distinct effects of prefrontal and parietal cortex inactivations on an accumulation of evidence task in the rat
Source: eLife. 2015 Apr 14;4:e05457. doi: 10.7554/eLife.05457 (PMC4392479; doi:10.7554/eLife.05457)
Supplement: Supplementary file 1. — Using the lme4 package to fit generalized-liner mixed models in R. This file contains the code (and links to our data) which shows how we used the lme4 package, in R, to fit generalized linear mixed models (GLMM). We also include the output of each of the GLMM we described in the main text. This allows the interested reader to regenerate our main results and also, by providing the data, allows the reader to perform additional statistical tests. DOI: http://dx.doi.org/10.7554/eLife.05457.024 [file elife05457s002.pdf]

Supp. File I: Using the lme4 package to fit Generalized Linear Mixed-Models in R

The following document describes, with embedded R code, the statistical tests and detailed results of those tests used to determine whether muscimol had a significant effect on behavior. We include links to the data sets that were analyzed in the paper, so that a reader may reproduce the results themselves. We use a logistic generalized-linear mixed-model (GLMM) approach to take advantage of our full data set. We use the `lme4` package for the GLMM and the `lattice` package for plotting. We used a logistic fit instead of using a non-linear 4-parameter sigmoid (as used for visualization in **Figure 3** in the results) because the logistic is more robust for fitting relatively small numbers of trials (as low as 50-100 for some individual experiments) and also can reliably fit severe effects of muscimol (as in **Figure 3–figure supplement 3B**, rats A066,A077) where the 4-parameter sigmoid is unconstrained for these data.

In order to run the R code in this document, you need to install R and the `lme4` and `lattice` packages. Required custom functions and setup variables can be added to your workspace by running the following line in your R console:

```
source("http://brodylab.princeton.edu/clicks/code/setup.R")
```

R commands are in Monaco font and can be copied and pasted into your R console.

Unilateral Inactivations

The columns of the data frame that are used for the statistics are as follows:

Column	Definition
**wr**	0 for went left, 1 for went right
**dC**	# Right - # Left Clicks
**side**	1 for right infusions, -1 for left infusions
**rat**	The name of the rat
**group**	1,2 or 3. Corresponding to the three experimental groups described in the results.

```
fof = read.table("http://brodylab.princeton.edu/clicks/data/fof_musc_uni.dat",
    colClass = c(NA, "integer", "integer", "factor", "factor", "factor"))
ppc = read.table("http://brodylab.princeton.edu/clicks/data/ppc_musc_uni.dat",
    colClass = c(NA, "integer", "integer", "factor", "factor", "factor"))
```

To statistically test whether there was a significant lateralized effect we specified a mixed-effect logistic regression as follows:

```
(fof.f = lmer(wr ~ dC * side + (dC|rat/side), data = fof, family = binomial,
    REML = F))
    plotdatafit(fof.f)
```

The model specifies an that *wr* is a function of *intercept* (implied), *dC, side* and their interaction as fixed effects and allows each rat to have 4 parameters which can each vary around the fixed effects, but sum (approximately) to zero. The notation (dC|rat/side) indicates that *side* is a within-subject condition. So each rat can vary from the fixed-effects with a shift, a change in slope, the degree of separation of left and right infusions, and a difference in the slope of left and right infusions. The `family = binomial` option specifies that the dependent variable is binomial and that the model should use a logistic linking function. The `REML = F` in the model specification indicates that the model should be fit using maximum likelihood (ML) estimation as opposed to reduced ML which fits the fixed effects first and then the random effects. The results are not changed if we use reduced ML.

```
## Generalized linear mixed model fit by the Laplace approximation
## Formula: wr ~ dC * side + (dC | rat/side)
##    Data: fof
##   AIC  BIC logLik deviance
##  4089 4153  -2034     4069
## Random effects:
##  Groups   Name        Variance Std.Dev. Corr
##  side:rat (Intercept) 2.292768 1.5142
##           dC          0.000275 0.0166   -0.088
##  rat      (Intercept) 0.174697 0.4180
##           dC          0.000245 0.0157   1.000
## Number of obs: 4521, groups: side:rat, 23; rat, 12
##
## Fixed effects:
##             Estimate Std. Error z value Pr(>|z|)
## (Intercept) -1.55843    0.46493   -3.35   0.0008 ***
## dC           0.06482    0.00832    7.80  6.4e-15 ***
## side1        3.37075    0.65122    5.18  2.3e-07 ***
## dC:side1     0.01464    0.01019    1.44   0.1509
## ---
```

There is a significant effect of side for unilateral FOF infusions (β_{side}=3.4 [2.1, 4.6] 95% CI, p=2.3e-07). The effect of *dC* on *wr* is also significant, meaning the rats are using the Clicks to guide their responses. Note the *dC:side1* interaction is not significant, suggesting that the left and right infusions did not have different effects on the slope of the logistics. Moreover, the sign of the infusion effect is consistent for every rat **(Figure 3–figure supplement 3B)**: there is more rightward responses for every rat during right than left infusions.

The PPC data was analyzed using the same model specification.

```
(ppc.f = lmer(wr ~ dC * side + (dC | rat / side), data = ppc, family = binomial,
    REML = F))
    plotdatafit(ppc.f)
```

```
## Generalized linear mixed model fit by the Laplace approximation
## Formula: wr ~ dC * side + (dC | rat/side)
##    Data: ppc
##    AIC   BIC logLik deviance
##  12489 12563  -6235    12469
## Random effects:
##  Groups   Name        Variance Std.Dev. Corr
##  side:rat (Intercept) 0.049400 0.2223
##           dC          0.000359 0.0190   0.634
##  rat      (Intercept) 0.065029 0.2550
##           dC          0.001033 0.0321   0.555
## Number of obs: 11498, groups: side:rat, 28; rat, 14
##
## Fixed effects:
##             Estimate Std. Error z value Pr(>|z|)
## (Intercept) -0.10581    0.09782   -1.08    0.279
## dC           0.08896    0.01045    8.51   <2e-16 ***
## side1        0.22174    0.09961    2.23    0.026 *
## dC:side1     0.00350    0.00846    0.41    0.679
## ---
## Signif. codes:  0 '***' 0.001 '**' 0.01 '*' 0.05 '.' 0.1 ' ' 1
##
## Correlation of Fixed Effects:
##          (Intr) dC     side1
## dC        0.509
## side1    -0.495 -0.190
## dC:side1 -0.239 -0.394  0.481
```

There is a significant effect of side for unilateral PPC infusions (β_{side}=0.22 [0.027, 0.42] 95% CI, p=0.026). This significant result may seem to contradict the *t*-test reported in the main text (*t*-test t_{13}=1.76, p>0.1). A *t*-test across 14 rats has substantially less statistical power than a GLMM across 11498 trials. The size of the effect is, of course, the same; the effect of infusions in the PPC is an order of magnitude smaller than the FOF effect. When we examine the data for each rat (**Figure 7-figure supplement 1**) it is clear that the effect of unilateral PPC infusion is nominal.

Analyzing the 3 groups of rats separately.

Group 1 (B115,T055,T057,T058,T061,T062)

The group 1 rats had cannula implanted in FOF and medial and lateral PPC (**Figure 3–figure supplement 1**). On the first day of infusion into PPC we observed a strong leftward bias (**Figure 3–figure supplement 2A**). All of these rats received a left PPC infusion on their first day. In hindsight, we realized that this introduced a confound, and in subsequent groups some rats received right and some rats received left infusions on each day. In total, we analyzed data from 7752 trials from 50 unilateral PPC infusions in the 6 group 1 rats. Because we collected so much data, the bias achieved statistical significant despite the small effect size (~10x less than FOF). For comparison the FOF data is comprised of 3090 trials from 24 unilateral FOF infusion sessions.

Group 1 FOF

```
fof1 = subset(fof, group == 1, drop = T)
fof1.f = lmer(wr ~ dC * side + (dC | rat / side), data = fof1, family = binomial,
    REML = F)
```

For group 1 FOF: (β_{side}=2.8 [1.5, 4.1] 95% CI, p=2.7e-05).

Group 1 PPC

```
ppc1 = subset(ppc, group == 1, drop = T)
ppc1.f = lmer(wr ~ dC * side + (dC | rat / side), data = ppc1, family = binomial,
    REML = F)
```

For group 1 PPC: (β_{side}=0.33 [0.045, 0.62] 95% CI, p=0.024).

Group 2 (A065,A066,A077,A078)

The group 1 rats had cannula implanted in both medial and lateral PPC (**Figure 3–figure supplement 1A**). Because of the close proximity of these cannulae we used 33 AWG injectors rather than the 28 AWG injectors used in the group 1 FOF cannula. Therefore, we developed three hypotheses about the transient effects of muscimol in group 1. First, that the thinner injectors in the PPC somehow led to different results (E.g. they caused more pressure and led to more scarring which blocked further effects). Second, that the rats adapted to the effects of muscimol, so even though PPC was being inactivated throughout the 11 infusion sessions, the rats' strategy had shifted from one that was PPC dependent to one that was PPC independent. Third, the effects in the first session happened by chance. While this third hypothesis may seem unlikely, it is important to remember that the Clicks task is a two-alternative forced choice task. One viable strategy is to simply respond to one side to collect half the reward. If the rats were disoriented because of the infusion procedure in general, they may have fallen back on a degenerate strategy, and by chance, they were biased to the same side. To determine which of the three hypotheses was correct, we repeated the experiment with group 2 rats, this time using identical cannula in FOF and PPC (**Figure 3–figure supplement 1B)**. We analyzed 2706 trials from 18 unilateral PPC infusion sessions and 1155 trials from 8 unilateral FOF infusion sessions in group 2 rats. We found significant effects in FOF but not in PPC as follows:

Group 2 FOF

```
fof2 = subset(fof, group == 2, drop = T)
fof2.f = lmer(wr ~ dC * side + (dC | rat / side), data = fof2, family = binomial,
    REML = F)
```

For group 2 FOF: (β_{side}=4.8 [2, 7.6] 95% CI, p=0.00084).

Group 2 PPC

```
ppc2 = subset(ppc, group == 2, drop = T)
ppc2.f = lmer(wr ~ dC * side + (dC | rat / side), data = ppc2, family = binomial,
    REML = F)
```

For group 2 PPC: (β_{side}=0.023 [-0.41, 0.46] 95% CI, p=0.92).

Note that even in the first PPC session for group 2 there were no effects (**Figure 3–figure supplement 2B**)

Group 3 (A060,A062,A083,A084)

Our PPC coordinates for group 1 and 2 (At 3.8 mm posterior and ~3 mm lateral to Bregma) were based on a rat atlas, the neural correlates of accumulation found at this location (T.D.H. et al.) and several published studies of rat PPC. However, some have suggested that PPC is more posterior: 4-6 mm posterior to Bregma (Kolb, 1987). Even though we had already used large doses that would likely have spread more than 3 mm away from the infusion site (**Figures 7B and 8**), for completeness we repeated the experiments again in another 4 rats (**Figure 3–figure supplement 1C)**. We analyzed 1105 trials from 12 unilateral PPC infusion sessions and 308 trials from 5 unilateral FOF infusion sessions in group 3 rats. We found significant effects in FOF but not in PPC as follows:

Group 3 FOF

```
fof3 = subset(fof, group == 3, drop = T)
fof3.f = lmer(wr ~ dC * side + (dC | rat / side), data = fof3, family = binomial,
    REML = F)
```

For group 3 FOF: (β_{side}=0.94 [0.31, 1.6] 95% CI, p=0.0036).

Group 3 PPC

```
ppc3 = subset(ppc, group == 3, drop = T)
ppc3.f = lmer(wr ~ dC * side + (dC | rat / side), data = ppc3, family = binomial,
    REML = F)
```

For group 3 PPC: (β_{side}=0.23 [-0.4, 0.87] 95% CI, p=0.47).

For all groups the coefficient for the FOF infusion side is significant. However, for the PPC, the infusion side coefficient is only significant in the Group 1 rats.

Bilateral Inactivations

For the bilateral inactivations, we fit a model that has trials labeled as whether they came from an infusion session or an isoflurane-only where rats received isoflurane, and were handled as if they were to receive an infusion, as on an infusion day, but did not receive an infusion. All bilateral data comes from group 2 rats. Here, we are looking for a non-lateralized change in slope. As we will see in the data, the bilateral infusions tended to cause a bias and a slope change, so we include both in the model. But the effect we are interested in is the slope - the interaction term between *inf* and *dC.*

Column	Definition
**rat**	The name of the rat
**wr**	0 for went left, 1 for went right
**inf**	0 for iso control, 1 for infusions
**dC**	# Right - # Left Clicks

```
fofbi = read.table("http://brodylab.princeton.edu/clicks/data/fof_musc_bi.dat",
    colClass = c(NA, "factor", "integer", "factor", "integer"))
ppcbi = read.table("http://brodylab.princeton.edu/clicks/data/ppc_musc_bi.dat",
    colClass = c(NA, "factor", "integer", "factor", "integer"))
fofbi.f = lmer(wr ~ dC * inf + (dC | rat / inf), data = fofbi, family = binomial,
    REML = F)
plotdatafit(fofbi.f, cond = "inf", w = 2, h = 2, clr1 = "black", clr2 = bi_clr)
```

```
ppcbi.f = lmer(wr ~ dC * inf + (dC | rat / inf), data = ppcbi, family = binomial,
    REML = F)
plotdatafit(ppcbi.f, cond = "inf", w = 2, h = 2, clr1 = "black", clr2 = bi_clr)
```

There was a significant decrease in the slope after bilateral FOF inactivations (**Figure 3–figure supplement 3a**, $\beta_{dC:inf}$=-0.032 [-0.059, -0.0055] 95% CI, p=0.018). There was also a significant decrease in the slope after bilateral PPC inactivations (**Figure 7–figure supplement 1a**, $\beta_{dC:inf}$=-0.014 [-0.026, -0.0023] 95% CI, p=0.02). Note that the coefficient for the PPC is half that of the FOF even though it is twice the dose of muscimol (150 ng/side vs. 75 ng/side)

Simultaneous bilateral fof and unilateral ppc

We analyzed the simultaneous infusion using the same model specification as the unilateral infusions. While we see both bias and slope effects, we are really interested in the bias here, as in the unilateral case.

Column	Definition
**wr**	0 for went left, 1 for went right
**dC**	# Right - # Left Clicks
**side**	-1 for left PPC, 1 for right PPC
**rat**	The name of the rat

Fetch the data from the Brody Lab server, fit the model, and plot the data.

```
bfup = read.table("http://brodylab.princeton.edu/clicks/data/bifof_unippc_musc.dat",
    colClass = c(NA, "integer", "integer", "factor", "factor"))
(bfup.f = lmer(wr ~ dC * side + (dC | rat / side), data = bfup, family = binomial,
    REML = F))
    plotdatafit(bfup.f, w = 2, h = 2)

## Generalized linear mixed model fit by the Laplace approximation
## Formula: wr ~ dC * side + (dC * side | rat)
##    Data: bfup
##   AIC  BIC logLik deviance
##  3196 3281  -1584     3168
## Random effects:
##  Groups Name        Variance Std.Dev. Corr
##  rat    (Intercept) 2.696067 1.6420
##         dC          0.000945 0.0307    0.956
##         side1       0.632019 0.7950   -0.985 -0.990
##         dC:side1    0.000253 0.0159    0.333  0.572 -0.452
## Number of obs: 3188, groups: rat, 4
##
## Fixed effects:
##              Estimate Std. Error z value Pr(>|z|)
## (Intercept)  -1.1815     0.8257   -1.43  0.15244
## dC            0.0593     0.0161    3.68  0.00024 ***
## side1         1.3257     0.4107    3.23  0.00125 **
## dC:side1      0.0130     0.0103    1.26  0.20941
## ---
```

```
## Signif. codes:  0 '***' 0.001 '**' 0.01 '*' 0.05 '.' 0.1 ' ' 1
##
## Correlation of Fixed Effects:
##          (Intr) dC     side1
## dC        0.907
## side1    -0.969 -0.918
## dC:side1  0.248  0.279 -0.312
```

There was a significant bias ipsilateral to the side of the PPC infusion after bilateral FOF + unilateral PPC inactivation (**Figure 9–figure supplement 1**, β_{side}=1.3 [0.52, 1.9] 95% CI, p=0.0012). This confirms that a) the inactivations of PPC were effective, ruling out methodological concerns for lack of PPC effects and b) the role of the PPC in the Clicks task can be revealed by silencing the activity in the FOF.
